# Fractal Analysis of Periodontal Tissues in Geriatric Patients With Different Periodontal Conditions: A Retrospective Study

**DOI:** 10.1111/odi.70389

**Published:** 2026-08-10

**Authors:** Dilek Ozkan Sen, Omer Ogutcen, Fatma Ucan Yarkac, Ibrahim Burak Yuksel, Muhammet Emin Arslan, Zeynep Tastan Eroglu

**Affiliations:** ^1^ Department of Periodontology, Faculty of Dentistry Necmettin Erbakan University Konya Turkey; ^2^ Department of Oral and Maxillofacial Radiology, Faculty of Dentistry Necmettin Erbakan University Konya Turkey

**Keywords:** aged, alveolar bone loss, fractals, panoramic radiography, periodontitis

## Abstract

**Objective:**

This study evaluated the relationship between fractal dimension (FD) values and periodontal status in geriatric patients using digital panoramic radiographs.

**Methodology:**

In this retrospective observational study, 147 elderly individuals were classified as periodontally healthy (*n* = 30), gingivitis (*n* = 32), Stage I periodontitis (*n* = 26), Stage II periodontitis (*n* = 14), Stage III periodontitis (*n* = 20), and Stage IV periodontitis (*n* = 25) according to the 2017 World Workshop Classification. FD values were calculated using the box‐counting method in ImageJ from standardized mandibular posterior trabecular bone regions (#35–36 and #45–46). Clinical periodontal parameters were recorded, and statistical analyses were performed using ANOVA and independent samples *t*‐test (*α* = 0.05).

**Results:**

No significant differences were observed among groups regarding age or gender distribution (*p* > 0.05), whereas clinical periodontal parameters and FD values differed significantly (*p* < 0.001). The highest FD values were observed in the healthy and gingivitis groups, with progressive decreases corresponding to periodontal disease severity. Gender‐related differences were detected in gingivitis and periodontitis groups (*p* < 0.05).

**Conclusion:**

FD values vary with periodontal disease progression in geriatric patients, suggesting that fractal analysis may support radiographic assessment of trabecular bone changes when interpreted alongside clinical findings.

## 1 Introduction

Periodontal disease, a prevalent inflammatory disorder predominantly affecting older individuals, leads to progressive loss of alveolar bone and can give rise to numerous systemic health complications (Al‐Harthi et al. 2022). With the global population steadily aging, the rising incidence of periodontitis in older individuals has become a pressing public health challenge, emphasizing the necessity for accurate diagnostic approaches to facilitate early detection and effective management (Filgueiras et al. 2023). Although conventional radiographic methods are widely applied in periodontal evaluations, they often fail to identify subtle alterations in trabecular bone architecture prior to significant bone resorption (Mishra et al. 2023). Accordingly, advanced image processing techniques, such as fractal analysis (FA), have emerged as promising tools to enhance the diagnostic efficacy of routine radiographs (Eser and Sarıbaş 2024; Updike and Nowzari 2008).

FA is a robust mathematical technique employed to quantify the complexity of structures by computing their fractal dimension (FD), which accurately represents morphological complexity and architectural intricacies. In the context of periodontology, FA has been widely applied to examine trabecular bone changes through panoramic and periapical radiographs, thereby providing a noninvasive and objective means of evaluating alveolar bone health (Updike and Nowzari 2008). Previous studies have consistently reported a decline in FD values with the progression of periodontitis, indicating a disruption in trabecular bone organization and an increase in bone porosity (Aktuna Belgin and Serindere 2020; Sener et al. 2015). While this technique holds considerable potential in differentiating healthy individuals from those experiencing varying degrees of periodontitis, its application within geriatric populations remains inadequately investigated (Yarkac et al. 2023).

As bone metabolism naturally slows with aging, accompanied by decreased bone turnover and an increased risk of osteoporotic alterations, evaluating periodontal bone loss in older adults becomes increasingly challenging. Therefore, investigating the potential of FA in geriatric populations may contribute to improving diagnostic accuracy. The complex association between aging, periodontal disease, and trabecular bone microarchitecture calls for more comprehensive research to validate FA as a trustworthy diagnostic tool in geriatric dentistry.

Therefore, this study aimed to evaluate the relationship between FD values and periodontal status in a cohort of elderly individuals and to assess the potential diagnostic utility of FA in detecting trabecular bone alterations associated with periodontal disease progression, thereby providing insight into its possible role in the diagnosis and monitoring of periodontal disease in aging populations.

It was hypothesized that FD values differ significantly among geriatric individuals with varying periodontal conditions and decrease progressively with increasing severity of periodontal disease. Accordingly, the null hypothesis (H0) stated that there is no significant difference in FD values among the groups, whereas the alternative hypothesis (H1) proposed that FD values decrease significantly as periodontal disease severity increases.

## 2 Material Methods

This retrospective study was conducted in accordance with the ethical principles outlined in the Declaration of Helsinki and reported following the STROBE guidelines for observational studies. Due to the retrospective design, the requirement for informed consent was waived by the institutional ethics committee. All radiographic data were anonymized prior to analysis to ensure patient confidentiality.

Ethical approval was obtained before the retrospective evaluation of panoramic radiographs and periodontal records archived in the Department of Periodontology. Initially, 180 panoramic radiographs were screened. After excluding patients with systemic diseases, medications affecting bone metabolism, non‐periodontal or endodontic lesions, fractures, or root canal treatments within the region of interest (ROI), a total of 147 participants were included in the study.

No formal a priori sample size calculation was performed due to the retrospective nature of the study. Therefore, the sample size was determined based on the availability of eligible radiographic records that met the predefined inclusion and exclusion criteria.

### 2.1 Periodontal Classification

The 2017 World Workshop Classification of Periodontal Diseases was used to categorize participants into six distinct categories based on their periodontal health status. In particular, the study population consisted of 30 healthy individuals (control group), 32 individuals with gingivitis (G), 26 individuals with Stage 1 periodontitis (S1‐P), 14 individuals with Stage 2 periodontitis (S2‐P), 20 individuals with Stage 3 periodontitis (S3‐P), and 25 individuals with Stage 4 periodontitis (S4‐P) (Loe et al. 1965).

The classification was determined using clinical and digital panoramic radiographic parameters, including probing pocket depth (PPD), clinical attachment level (CAL), and radiographic bone loss (RBL).

Individuals with probing depths < 3 mm, clinical attachment loss absent, and bleeding on probing (BOP) < 10% were categorized as periodontally healthy (Loe and Silness 1963). Gingivitis cases were identified by BOP > 10% without attachment loss. Periodontitis cases were classified based on the severity of CAL, in line with the 2017 World Workshop Classification of Periodontal and Peri‐Implant Diseases and Conditions. CAL measurements of 1–2 mm were defined as Stage I, 3–4 mm as Stage II, and greater than 4 mm as Stages III or IV. In addition, patients with more than 30% of affected teeth were categorized as having generalized periodontitis. To ensure homogeneity of the study population and enable consistent comparison of bone changes across different stages, only patients diagnosed with generalized periodontitis were included. Complexity factors (e.g., furcation involvement and tooth mobility) were also evaluated when available to support stage assignment (Loe et al. 1965). In cases where CAL data were limited or incomplete, RBL was assessed using digital panoramic radiographs, which were obtained from the institutional digital archive. Radiographic staging was based on the percentage of bone loss relative to root length, as recommended in the 2017 classification guidelines. All clinical measurements were performed by a single experienced periodontist (F.U.Y).

Periodontal health status was rigorously evaluated by assessing both clinical and radiographic parameters across all individuals. To determine supragingival plaque accumulation, the full‐mouth plaque index (PI) (Soylu et al. 2021) was calculated by examining six sites on each tooth. Visual assessment of gingival inflammation was conducted using the gingival index (GI) (Soltani et al. 2021), which categorizes inflammation severity on a scale from 0 to 3.

This scale considers the color, texture, edema, and spontaneous bleeding of both the marginal and papillary gingiva, with the average score being recorded. PPD and CAL were measured at six sites per tooth, including the mesio‐buccal, mid‐buccal, disto‐buccal, mesio‐lingual, mid‐lingual, and disto‐lingual sites, using a periodontal probe (UNC 15, Hu‐Friedy, Chicago, IL, USA). The measurements were rounded to the nearest millimeter. BOP was also recorded after measuring the probing depth at each site, and the percentage of sites with bleeding was calculated (Soltani et al. 2021).

### 2.2 Radiographic Data Acquisition and Image Processing

#### 2.2.1 Image Acquisition

Panoramic radiographs were obtained using the Planmeca ProOne (Planmeca Oy, Helsinki, Finland) device, operating at 68 kVp, 7 mA, and a 10‐s exposure time, with a single operator performing the procedure.

During panoramic image acquisition, patients were instructed to stand in an upright position with the Frankfort horizontal plane parallel to the floor and the midsagittal plane aligned vertically with the positioning light beam. The chin rest, bite block, and forehead support were used to stabilize the head and minimize movement. Patients were asked to gently bite on the bite block and remain still while keeping their lips closed and tongue against the palate throughout the 10‐s exposure period. All panoramic images were acquired using the same device and were captured by the same experienced operator to ensure consistency.

The radiographic images were processed and analyzed using Romexis software (Planmeca, Helsinki, Finland). During the initial stage of image optimization, the software was applied to ensure consistent brightness, contrast, and filtration settings. The resulting raw data images were then analyzed to maintain data accuracy and standardization.

#### 2.2.2 Image Processing

In the course of image analysis, panoramic radiographic images were adjusted to a resolution of 1024 × 499 pixels and a density of 600 dpi through the use of PhotoScape X Pro (MOOII Tech, South Korea). To maintain data integrity and ensure optimal quality, the images were saved in TIF (Tagged Image File) format. The resizing procedure was applied uniformly to all radiographs to ensure methodological standardization and consistent ROI selection throughout the dataset. Although image interpolation during resizing may potentially influence trabecular pattern representation and FD measurements, identical preprocessing parameters were applied to all images to minimize systematic bias.

For FA, the ImageJ v1.54 software (National Institutes of Health, Bethesda, MD, USA) was obtained from the official website (https://imagej.nih.gov/ij/download.html) and applied to the dataset. To improve the consistency of the measurements, 20% of the dataset was randomly selected and reassessed by two independent researchers (I.B.Y. and M.E.A.) at a 15‐day interval using the same image processing protocol.

The radiographic examination was conducted using a PC with an AMD Ryzen 5 processor running Windows 11 Professional, equipped with 8 GB RAM, a 15.6‐in. flat‐panel color display with a resolution of 1920 × 1080 pixels, and a Geforce RTX 3050 graphics card (Lenovo 15.6″ FHD 120 Hz).

Image analysis was carried out using ImageJ software, employing the box‐counting method introduced by White and Rudolph to calculate the FD (White and Rudolph 1999). After importing the panoramic image into the software, ROIs were selected in accordance with predefined anatomical and methodological criteria. These areas were chosen for their consistent trabecular architecture, morphological stability, and minimal interference from overlapping anatomical structures such as the mandibular canal, mental foramen, or root outlines. Moreover, their location in posterior mandibular sites reflects functional masticatory zones, enhancing clinical relevance.

Specifically, ROI1 was defined as a 25 × 25 pixel square between the apical regions of teeth #35 and #36, while ROI2 was positioned similarly between teeth #45 and #46 (Figure 1). In cases where the interproximal area did not precisely meet the spatial criteria, the ROI was carefully adjusted to the nearest appropriate pixel location to preserve consistency across subjects.

**FIGURE 1:**
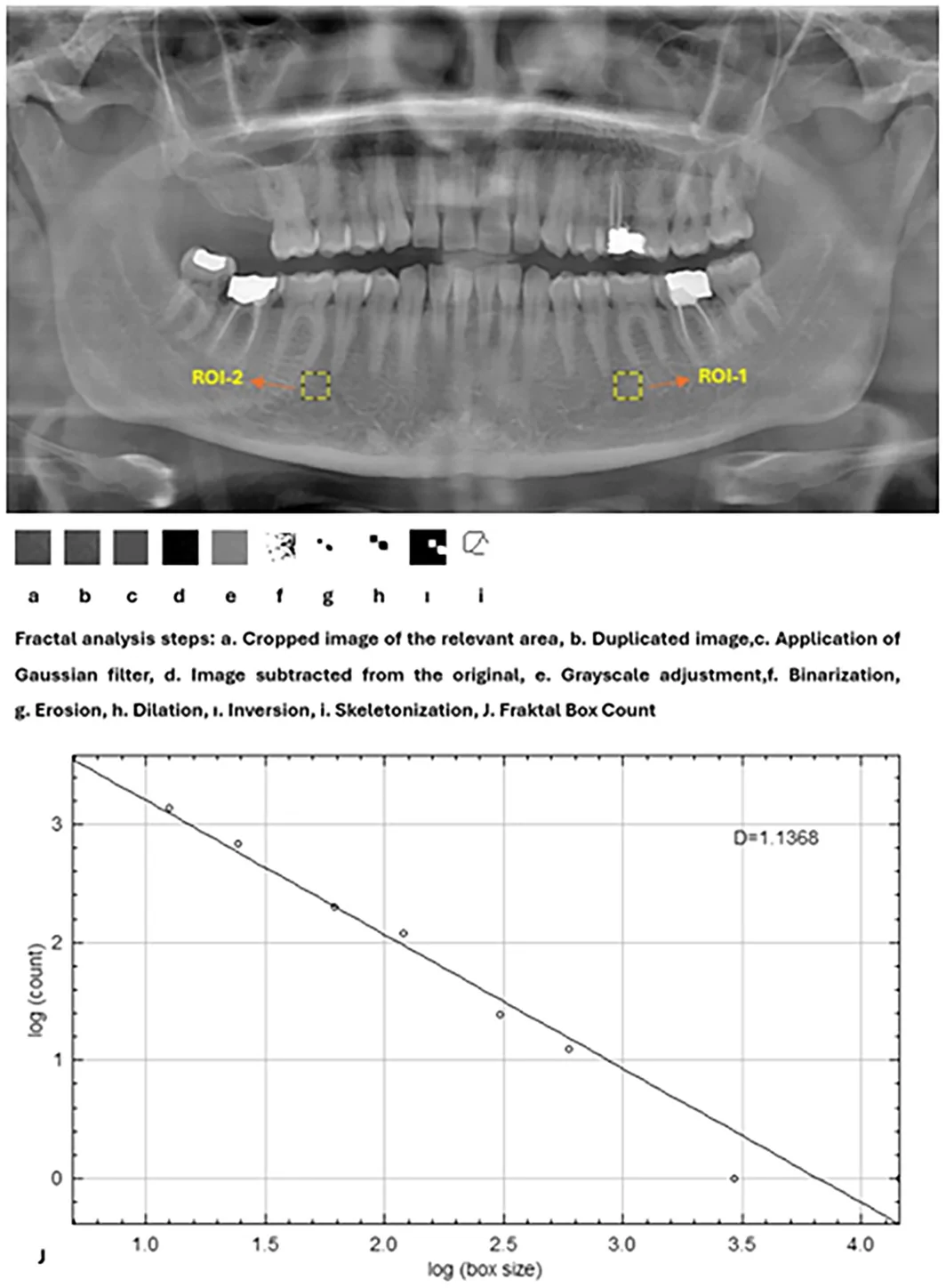
Workflow of fractal analysis and region of interest (ROI) selection. Digital panoramic radiographs were imported into ImageJ software, and standardized preprocessing steps were applied. Square ROIs (25 × 25 pixels) were selected from the interdental trabecular bone between the mandibular first molar and second premolar regions (#35–36 and #45–46). Fractal dimension (FD) values were calculated using the box‐counting method.

##### 2.2.2.1 ROI Processing and FD Calculation

All selected ROIs were digitally cropped and duplicated. To eliminate uneven background illumination, a Gaussian blur filter (radius = 35) was applied to the duplicated images. The filtered image was then subtracted from the original image, and a constant grayscale value of 128 was added to normalize brightness. Following this step, the images were binarized using a fixed threshold of 128.

To reduce noise and isolate the trabecular pattern, erosion and dilation operations were applied sequentially. The processed images were subsequently skeletonized, reducing trabecular structures to a single‐pixel thickness. In the final skeletonized images, white pixels represented trabecular bone, while black pixels corresponded to marrow spaces.

FD values were calculated using the box‐counting algorithm. Grid sizes of 2, 3, 4, 6, 8, 12, 16, 32, and 64 pixels were applied to each ROI, and the number of boxes intersecting the trabecular structure was recorded. FD values were derived from the slope of the regression line on a log–log plot of box size versus box count. Higher FD values indicate greater structural complexity of the trabecular bone.

### 2.3 Statistical Analysis

Data analysis was carried out using SPSS software version 21.0 (IBM Corp, Armonk, NY, USA). To evaluate intra‐observer reliability, the intraclass correlation coefficient (ICC) analysis was employed. Descriptive statistics were expressed as mean ± standard deviation for continuous variables and as frequencies and percentages for categorical variables. The normality of continuous data was assessed using the Kolmogorov–Smirnov test.

Differences in demographic variables and categorical data among groups were analyzed using the chi‐square test. Comparisons of clinical periodontal parameters and FD values among the study groups were performed using one‐way analysis of variance (ANOVA), followed by Bonferroni post hoc tests for pairwise comparisons. Differences in FD values according to gender were evaluated using the independent samples *t*‐test. A Type I error rate of 5% was accepted, and statistical significance was set at *p* < 0.05.

## 3 Results

### 3.1 Demographic Data

The participants' ages ranged from 65 to 80 years across all groups.

There were no statistically significant differences in mean age among the groups (*p* = 0.762). Similarly, gender distribution did not differ significantly between the groups (*p* = 0.347). However, a statistically significant difference was observed in the frequency of dental visits and tooth brushing among the groups (*p* < 0.05). Participants in the healthy group demonstrated the highest frequency of regular biannual dental visits and twice‐daily tooth brushing, whereas individuals with Stage IV periodontitis showed the lowest oral hygiene frequency and less regular dental attendance (Table 1).

**TABLE 1:** Patient demographics and habits.

Parameters	Healthy (*n* = 30)	Gingivitis (*n* = 32)	Stage I periodontitis (*n* = 26)	Stage II periodontitis (*n* = 14)	Stage III periodontitis (*n* = 20)	Stage IV periodontitis (*n* = 25)	*p*
Age, years (mean ± SD)	65.80 ± 1.66 (65–69)	65.97 ± 3.09 (66–72)	65.62 ± 2.57 (65–70)	66.00 ± 1.56 (65–69)	66.90 ± 6.66 (65–80)	66.80 ± 3.40 (65–74)	**0.762**
Gender, *n* (%)							**0.347**
Male	13 (43.3)	13 (40.6)	12 (46.2)	6 (42.9)	14 (70.0)	14 (56.0)	
Female	17 (56.7)	19 (59.4)	14 (53.8)	8 (57.1)	6 (30.0)	11 (44.0)	
Dental visit frequency, *n* (%)							**0.017**
Visits due to complaints	11 (36.7)	23 (71.9)	18 (69.2)	6 (42.9)	14 (70.0)	16 (64.0)	
Every 6 months	14 (46.7)	2 (6.3)	4 (15.4)	5 (35.7)	6 (30.0)	5 (20.0)	
Once a year	5 (16.7)	7 (21.9)	4 (15.4)	3 (21.4)	0 (0.0)	4 (16.0)	
Tooth‐brushing frequency, *n* (%)							**0.001**
Rarely	3 (10.0)	10 (31.3)	11 (42.3)	3 (21.4)	6 (30.0)	15 (60.0)	
Once daily	14 (46.7)	10 (31.3)	9 (34.6)	6 (42.9)	12 (60.0)	10 (40.0)	
Twice daily	13 (43.3)	12 (37.5)	6 (23.1)	5 (35.7)	2 (10.0)	0 (0.0)	

*Note:* All data except age are presented as *n* (%). Bold *p* values indicate statistically significant differences between groups. Age comparisons were performed using one‐way ANOVA, whereas categorical variables were analyzed using the chi‐square test.

### 3.2 Clinical Periodontal Measurements and FD Values

The clinical periodontal measurements and FD values for each group are summarized in Table 2. Statistically significant differences were observed among the groups for all clinical periodontal parameters and FD measurements (*p* < 0.05). An increasing trend in PPD values was observed with increasing periodontal disease severity, with the highest mean PPD detected in the Stage IV periodontitis group (3.40 ± 0.76 mm; *p* < 0.001). Similarly, PI, GI, and BOP values increased progressively across the periodontal disease groups and reached their highest levels in the Stage IV periodontitis group (*p* < 0.001).

**TABLE 2:** Clinical periodontal measurements and FD values (mean ± standard deviation).

Periodontal parameters	Healthy (*n* = 30)	Gingivitis (*n* = 32)	Stage I periodontitis (*n* = 26)	Stage II periodontitis (*n* = 14)	Stage III periodontitis (*n* = 20)	Stage IV periodontitis (*n* = 25)	*p*
PPD (mm)	2.30 ± 0.17	2.47 ± 0.29	2.81 ± 0.31	3.00 ± 0.02	3.28 ± 0.61	3.40 ± 0.76	**< 0.001**
PI	1.00 ± 0.64	1.78 ± 0.60	1.81 ± 0.69	2.00 ± 0.67	1.75 ± 0.63	2.52 ± 0.58	**< 0.001**
GI	0.83 ± 0.46	1.72 ± 0.68	1.69 ± 0.54	1.71 ± 0.72	1.75 ± 0.63	1.96 ± 0.35	**< 0.001**
BOP (%)	3.90 ± 3.49	46.84 ± 20.21	64.81 ± 28.24	57.29 ± 28.39	54.40 ± 29.40	59.84 ± 31.31	**< 0.001**
FD (35–36)	1.13 ± 0.08	1.14 ± 0.09	1.05 ± 0.04^a^, ^b^	1.06 ± 0.04^a^, ^b^	1.07 ± 0.05^a^, ^b^, ^c^	1.07 ± 0.03^a^, ^b^, ^c^	**< 0.001**
FD (45–46)	1.11 ± 0.09	1.11 ± 0.06	1.05 ± 0.04^a^, ^b^	1.06 ± 0.04	1.09 ± 0.03	1.06 ± 0.04	** 0.002**

*Note:* Intergroup comparisons were performed using one‐way ANOVA followed by Bonferroni post hoc tests. Bold *p* values indicate statistically significant differences.

Abbreviations: BOP, bleeding on probing; FD (35–36), fractal dimension of the region between teeth #35 and #36; FD (45–46), fractal dimension of the region between teeth #45 and #46; GI, gingival index; PI, plaque index; PPD, probing pocket depth.

^a^ Significantly different from the healthy group (*p* < 0.05).

^b^ Significantly different from the gingivitis group (*p* < 0.05).

^c^ Significantly different from the Stage II periodontitis group (*p* < 0.05).

FD measurements revealed significant intergroup differences in both mandibular posterior regions. The healthy and gingivitis groups demonstrated the highest FD values in the region between teeth #35 and #36 (1.13 ± 0.08 and 1.14 ± 0.09, respectively), whereas lower FD values were observed in all periodontitis groups (*p* < 0.001). Post hoc analyses demonstrated that FD values in the healthy and gingivitis groups were significantly higher than those observed in periodontitis groups. A similar pattern was identified in the region between teeth #45 and #46, where the healthy and gingivitis groups also exhibited higher FD values compared with the periodontitis groups (*p* = 0.002). The distribution and progressive reduction of FD values across periodontal groups are presented in Figures 2 and 3.

**FIGURE 2:**
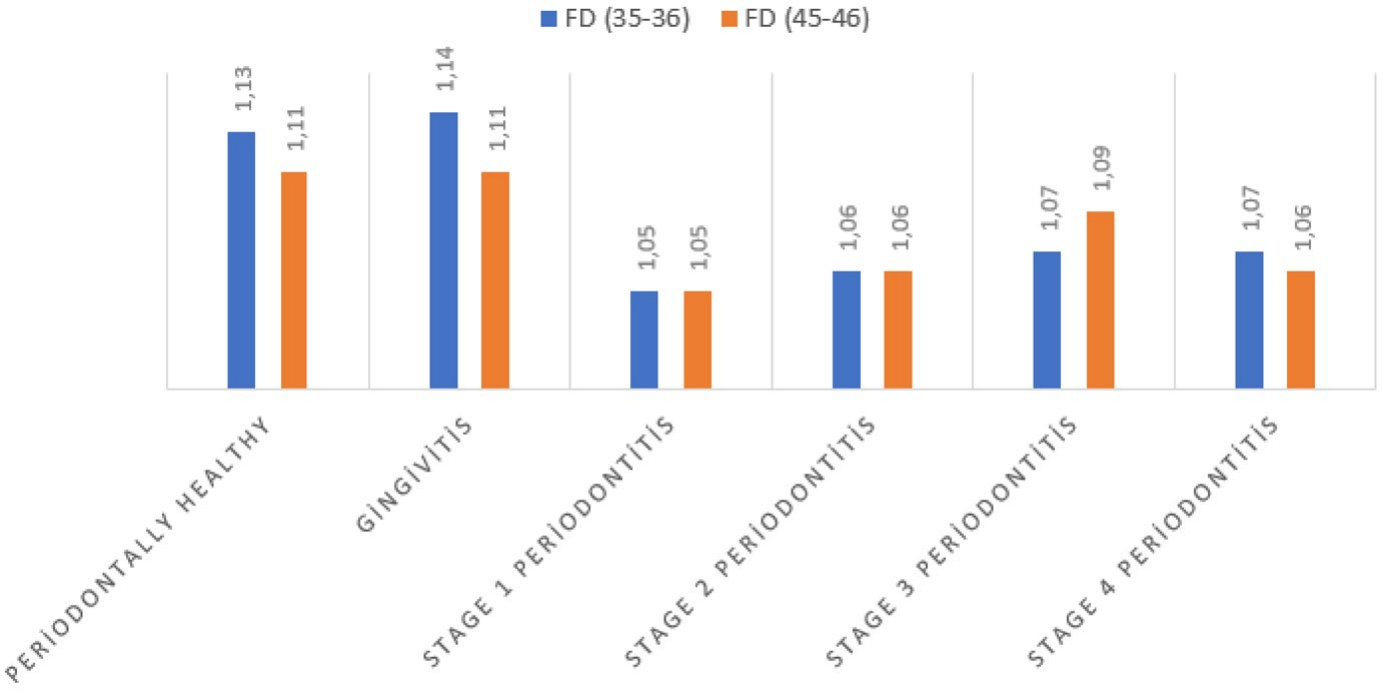
Distribution of fractal dimension (FD) values across periodontal groups. Mean FD values measured in the mandibular posterior regions (#35–36 and #45–46) are shown according to periodontal diagnosis. Higher FD values were observed in the healthy and gingivitis groups, whereas lower values were detected in the periodontitis groups.

**FIGURE 3:**
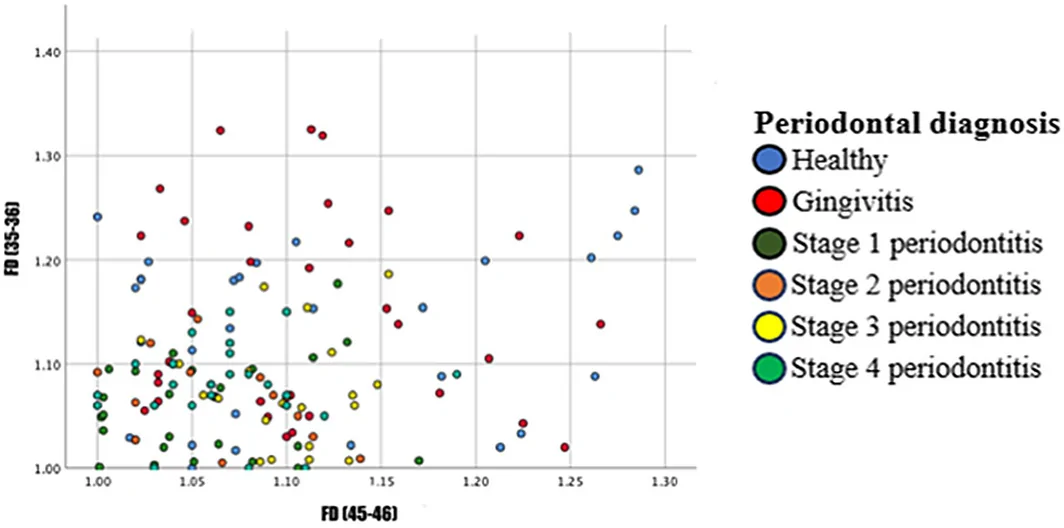
Scatter plot of FD values according to periodontal diagnosis. Scatter distribution of FD values for regions #35–36 and #45–46 across different periodontal stages. A decreasing trend in FD values is observed with increasing periodontal disease severity.

### 3.3 Gender‐Based Differences in FD Values

Gender‐related differences in FD values were evaluated across periodontal groups (Table 3). In the periodontal health group, no statistically significant differences in FD values were observed between males and females in either the region between teeth #35 and #36 or the region between teeth #45 and #46 (*p* = 0.803 and *p* = 0.684, respectively).

**TABLE 3:** Gender‐based comparison of FD values (mean ± standard deviation).

Periodontal status	Gender	FD (35, 36) (mean ± SD)	*p*	FD (45–46) (mean ± SD)	*p*
Periodontal Health	Female (*n* = 17)	1.13 ± 0.09	0.803	1.12 ± 0.09	0.684
	Male (*n* = 13)	1.12 ± 0.07		1.10 ± 0.09	
Gingivitis	Female (*n* = 19)	1.11 ± 0.09	0.007	1.10 ± 0.06	0.890
	Male (*n* = 13)	1.20 ± 0.07		1.11 ± 0.07	
Periodontitis	Female (*n* = 39)	1.05 ± 0.03	0.001	1.06 ± 0.03	0.026
	Male (*n* = 47)	1.08 ± 0.05		1.08 ± 0.05	

*Note:* Comparisons between genders were performed using the independent samples *t*‐test. Bold *p* values indicate statistically significant differences between groups.

Abbreviations: FD (35, 36), fractal dimension of the region between teeth #35 and #36; FD (45–46), fractal dimension of the region between teeth #45 and #46.

In the gingivitis group, males demonstrated significantly higher FD values than females in the region between teeth #35 and #36 (*p* = 0.007), whereas no significant difference was detected in the region between teeth #45 and #46 (*p* = 0.890).

In the periodontitis group, male participants exhibited significantly higher FD values than female participants in both mandibular posterior regions (#35–36 and #45–46) (*p* = 0.001 and *p* = 0.026, respectively).

## 4 Discussion

To the best of our knowledge, this study is the first to explore the link between periodontal health and trabecular bone microarchitecture in elderly individuals using FA on digital panoramic radiographs. Our findings demonstrate a clear reduction in FD values as periodontal disease advances, indicating heightened bone porosity and diminished trabecular complexity at later stages. These outcomes are consistent with earlier studies that have documented a connection between decreased FD values and more severe alveolar bone loss, further supporting the application of FA as an effective method for assessing trabecular changes in periodontal disease (Updike and Nowzari 2008; Aktuna Belgin and Serindere 2020; Sener et al. 2015; Loe et al. 1965; Soltani et al. 2021). Although the differences in FD values between groups appear small, they may still be clinically relevant, as FD reflects microarchitectural alterations in trabecular bone microarchitecture. Even minor reductions may indicate subtle structural deterioration and support the detection of trabecular changes in clinical practice. The lack of a statistically significant difference between Stages III and IV periodontitis may reflect a plateau effect in advanced disease and relatively limited subgroup sizes.

Although FD analysis has yet to be thoroughly explored in periodontitis patients, studies conducted by Shrout et al. and Sener et al. have demonstrated its potential to effectively differentiate between healthy and diseased individuals by quantifying trabecular bone degradation. Likewise, research carried out by Yarkac et al. and Stødle et al. revealed markedly lower FD values in individuals with Stage III/IV periodontitis, thereby emphasizing the applicability of FA in detecting alveolar bone loss (Yarkac et al. 2023; Stødle et al. 2023). Similarly, Korkmaz et al. and Dosdogru et al. documented a progressive decline in FD values as periodontal disease severity escalates, further supporting the use of FA for evaluating trabecular bone deterioration (Dosdogru et al. 2025; Korkmaz et al. 2023). Although previous studies have reported conflicting findings regarding the relationship between periodontal disease severity and FD values, some investigations have identified a significant correlation, whereas others have failed to detect notable differences (Tambasco et al. 2010; Wang et al. 2018; Arora et al. 2020; Kyllar et al. 2013). In the present study, FD values were significantly lower in periodontitis patients, confirming the detrimental impact of periodontal disease on trabecular bone architecture. The observed progressive decline in FD values also highlights the potential of FA as an objective marker of bone deterioration. However, it should be acknowledged that FD values may not directly correspond to the severity of periodontal conditions but rather reflect broader structural alterations within the periodontium, which could arise from differences in study design, sample sizes, or statistical methodologies. Moreover, FD may be particularly useful in early‐stage disease evaluation because it can detect subtle trabecular changes before more pronounced deterioration becomes apparent (Soğur and Baksı 2014; İrer et al. 2017; Güleç et al. 2019).

It is particularly important to investigate the relationship between FD and periodontal disease in elderly populations. Aging affects bone metabolism by decreasing turnover and increasing porosity, which may confound FD interpretation. These age‐related alterations may complicate FD interpretation, as both aging and periodontitis disrupt trabecular bone structure in a similar manner. In fact, previous studies have demonstrated that age‐induced changes in trabecular microarchitecture are a natural occurrence, which complicates the distinction between bone loss caused by aging and resorption caused by periodontitis (Giordano et al. 2016; Stauber and Müller 2006). These findings suggest that FA may serve not only as a descriptive imaging tool but also as a clinically relevant parameter for assessing the extent of trabecular bone deterioration associated with periodontal disease progression. Consequently, these findings highlight the importance of a comprehensive periodontal assessment in older individuals. Furthermore, the growing prevalence of periodontal disease in geriatric populations emphasizes the need for reliable diagnostic and monitoring methods (Shetty et al. 2019; Yeun et al. 2022; Ramos‐Gómez 2014).

Our study also revealed significant gender‐related differences in FD values, with males demonstrating higher FD values in both gingivitis and periodontitis groups. These findings may be associated with sex‐related differences in bone metabolism, hormonal regulation, inflammatory response, and trabecular remodeling patterns. In particular, postmenopausal hormonal alterations and age‐related reductions in bone mineral density may contribute to increased trabecular deterioration in elderly female individuals, potentially influencing FD measurements.

In addition, sex‐related variations in inflammatory response and periodontal tissue breakdown may partially explain the observed differences between males and females. Clinically, these findings suggest that gender should be considered when interpreting trabecular bone alterations and FD measurements in elderly patients with periodontal disease.

In addition, the significant differences observed in oral hygiene behaviors and dental attendance patterns among the groups may have acted as important confounding factors influencing both clinical periodontal parameters and FD values. Individuals with irregular dental visits and lower tooth brushing frequency, particularly in advanced periodontitis groups, may exhibit increased inflammatory burden and accelerated trabecular bone deterioration, potentially contributing to lower FD measurements. Future research should explore the role of systemic health and hormonal influences on trabecular bone properties in elderly populations where these factors are more pronounced (Branco‐de‐Almeida et al. 2012; Yadav et al. 2010; Li et al. 2020). In addition, our study observed notable differences in oral hygiene habits and the frequency of dental visits across periodontal groups, with the Stage IV periodontitis group having the lowest dental visit frequency (6.8%) and the highest prevalence of infrequent tooth brushing (31.3%). These findings further emphasize the importance of preventive dental care in preserving trabecular bone integrity and reducing periodontal deterioration in elderly individuals (Li et al. 2020; Guiglia et al. 2013; Karayürek et al. 2024; Aydın et al. 2020; Büyük and Arsan 2023).

This study has several limitations that should be acknowledged. One of the main limitations of the study is its cross‐sectional design, which inherently limits our ability to track changes in FD values over time, limiting the evaluation of longitudinal changes in trabecular bone structure. Therefore, future research should focus on longitudinal studies to better understand the dynamic relationship between periodontal disease and trabecular bone architecture. In addition, the relatively limited sample size may restrict the generalizability of the findings. Digital panoramic radiographs are commonly used for periodontal diagnosis because they are practical and accessible. However, they do not provide the spatial resolution achieved by more advanced imaging methods, such as cone‐beam computed tomography (CBCT) or micro‐computed tomography (Micro‐CT) (Tosun et al. 2021; Tomofuji et al. 2016; Kindler et al. 2017). These limitations may have influenced the accuracy of FD measurements and should therefore be considered when interpreting the results.

Several methodological considerations related to FD assessment should also be acknowledged. In our study, a high degree of interobserver consistency was observed in ROI selection. However, the influence of ROI selection on FD measurements requires further standardization and investigation. Developing standardized protocols would not only improve measurement reliability but also ensure consistency across future studies. In addition, the absence of a statistically significant difference in FD values between Stage III and IV periodontitis may indicate a nonlinear progression of trabecular bone loss at advanced stages. It is possible that bone microarchitecture reaches a plateau beyond a certain disease threshold, particularly in elderly individuals. Limited subgroup sizes, inter‐individual biological variability, or localized differences in trabecular patterns may also contribute to this observation. Therefore, the findings should be interpreted with caution, and future studies incorporating larger sample sizes, longitudinal designs, and high‐resolution imaging techniques are warranted to further elucidate this phenomenon.

## 5 Conclusion

In conclusion, our study pointed to the considerable potential of FA as a valuable adjunct to conventional periodontal diagnostics, particularly in evaluating trabecular bone changes in elderly patients. The pronounced decrease in FD values with advancing periodontal disease severity indicated that FA may contribute to the early detection and continuous monitoring of periodontal bone loss, thereby significantly enhancing clinical decision‐making and patient management.

## Author Contributions

**Muhammet Emin Arslan:** conceptualization, investigation, funding acquisition, writing – original draft, methodology. **Omer Ogutcen:** conceptualization, investigation, writing – original draft, writing – review and editing, validation, methodology, visualization, project administration, formal analysis, software. **Zeynep Tastan Eroglu:** conceptualization, investigation, funding acquisition, writing – original draft. **Ibrahim Burak Yuksel:** conceptualization, investigation, funding acquisition, writing – original draft, methodology, validation, visualization. **Dilek Ozkan Sen:** conceptualization, investigation, writing – review and editing, visualization, writing – original draft, methodology, validation, project administration, formal analysis, software, data curation, supervision, resources. **Fatma Ucan Yarkac:** conceptualization, investigation, funding acquisition, writing – original draft, writing – review and editing, visualization, validation, methodology, project administration.

## Conflicts of Interest

The authors declare no conflicts of interest.

## Supporting information


**Data S1:** STROBE checklist.

## Data Availability

The data that support the findings of this study are available on request from the corresponding author. The data are not publicly available due to privacy or ethical restrictions.
