# Supplementary material for: Fractal Analysis of Periodontal Tissues in Geriatric Patients With Different Periodontal Conditions: A Retrospective Study
Source: Oral Dis. 2026 Aug 10;32(10):3303–11. doi: 10.1111/odi.70389 (PMC13768897; doi:10.1111/odi.70389)
Supplement: Supplementary file 1 [file ODI-32-3303-s001.docx]

# STROBE Checklist

| Section/Topic | Item No | Recommendation |  |
| --- | --- | --- | --- |
| Title and Abstract | 1a | Indicate the study’s design with a commonly used term in the title or abstract |  |
|  | 1b | Provide an informative and balanced summary of what was done and what was found |  |
| Introduction | 2 | Explain the scientific background and rationale for the investigation |  |
|  | 3 | State specific objectives, including any prespecified hypotheses |  |
| Methods | 4 | Present key elements of study design early in the paper |  |
|  | 5 | Describe the setting, locations, and relevant dates |  |
|  | 6a | Give the eligibility criteria and the sources and methods of selection of participants |  |
|  | 7 | Clearly define all outcomes, exposures, predictors, potential confounders, and effect modifiers |  |
|  | 8 | For each variable of interest, give sources of data and details of methods of assessment |  |
|  | 9 | Describe any efforts to address potential sources of bias |  |
|  | 10 | Explain how the study size was arrived at |  |
|  | 11 | Explain how quantitative variables were handled in the analyses |  |
|  | 12a | Describe all statistical methods, including those used to control for confounding |  |
|  | 12b | Describe any methods used to examine subgroups and interactions |  |
|  | 12c | Explain how missing data were addressed |  |
|  | 12d | If applicable, describe analytical methods taking account of sampling strategy |  |
|  | 12e | Describe any sensitivity analyses |  |
| Results | 13a | Report numbers of individuals at each stage of study |  |
|  | 13b | Give reasons for non-participation at each stage |  |
|  | 13c | Consider use of a flow diagram |  |
|  | 14a | Give characteristics of study participants |  |
|  | 14b | Indicate number of participants with missing data for each variable |  |
|  | 15 | Report numbers of outcome events or summary measures |  |
|  | 16a | Give unadjusted estimates and, if applicable, confounder-adjusted estimates |  |
|  | 16b | Report category boundaries when continuous variables were categorized |  |
|  | 16c | If relevant, translate estimates of relative risk into absolute risk |  |
|  | 17 | Report other analyses done (subgroups, interactions, sensitivity analyses) |  |
| Discussion | 18 | Summarize key results with reference to study objectives |  |
|  | 19 | Discuss limitations of the study |  |
|  | 20 | Give a cautious overall interpretation of results |  |
|  | 21 | Discuss the generalizability (external validity) of the study results |  |
| Other Information | 22 | Give the source of funding and the role of the funders |  |
